# Ptyctimous Mites (Acari, Oribatida) of Peru with the Description of an Extraordinary New Phthiracaroid Mite from the Peruvian Andes †

**DOI:** 10.3390/ani13152403

**Published:** 2023-07-25

**Authors:** Wojciech Niedbała, Zbigniew Adamski, Ronald Laniecki, Wojciech L. Magowski

**Affiliations:** 1 Department of Animal Taxonomy and Ecology, Faculty of Biology, Adam Mickiewicz University, 61-614 Poznan, Poland; 2 Department of Animal Physiology and Developmental Biology, Laboratory of Electron and Confocal Microscopy, Faculty of Biology, Adam Mickiewicz University, 61-614 Poznan, Poland

**Keywords:** ptyctimous mites, *Protophthiracarus*, neotropical realm, neotrichy, new species

## Abstract

**Simple Summary:**

In this work, a new species of ptyctimous mite (“box mite”)—*Protophthiracarus afthonos*—is described and illustrated. It is exceptional by its very rich body setation—bigger than in any other known member of the ptyctimous oribatids. The new species was found in a forest soil sample from the Andes in Peru. The discovery confirms the uniqueness of the montane arthropod fauna of South America. The biogeographic distribution of ptyctimous oribatids in Peru is summarized and supplied with a key to the species of Peruvian fauna.

**Abstract:**

*Protophthiracarus afthonos* sp. nov. is described and illustrated using line drawings, transmitted light and SEM imaging. It is characterized by an extraordinary richness of notogastral setae (ca. 166 pairs) that has been previously unseen among phthiracaroid mites. The species originates from the material collected from the litter of primary forest in the Peruvian Andes. The genus *Protophthiracarus* is well represented in the Neotropical Region. Many species of ptyctimous mites have been found in Peru, representing both widespread and endemic biogeographic elements. Among a total of 37 species, 20 from Peru have been described for the first time. Currently, the ptyctimous fauna consists of 12 endemite, 11 neotropical, 4 semicosmopolitan and 9 pantropical biogeographic elements.

## 1. Introduction

Ptyctimous mites (Acari: Oribatida) are doubtlessly one of the best-known taxonomic groups of oribatid mites worldwide. Despite so many new species being described from all geographic regions of the world, new habitats are being explored, yielding yet more new taxa.

Peru is covered by a diverse range of habitats, from the Amazon rainforest in the east, to the high Andes mountains in the west. These habitats support a remarkable array of plant and animal life, including mites. Currently, there are three land domains of faunas: Amazon (with Amazon, Yungas, Pacific, Equatorial and Páramo provinces), Chaco and Andean-Patagonian (with Puno, Deserts and Mountain Steppes provinces). Knowledge of the distribution of most of invertebrate groups among those biogeographic units is fragmentary at best, if not nonexistent.

Peru is occupied by various vegetation units, namely mixed zones with evergreen wet tropical forests, mountainous tropical and subtropical wet forests, dry equatorial climate forests, savannahs and semideserts, trees and shrubs, grasslands, cushionplants and shrubs, and moss and lichens [1].

In this paper, we report a new, endemic species of the genus *Protophthiracarus* (Phthiracaroidea) from the Peruvian Andes that has a high, previously unseen number of notogastral setae, with the main purpose being to supply a description and images of this new, unusual taxon. In addition, our objective is to showcase the current picture of the ptyctimous mite fauna of Peru.

## 2. Materials and Methods

Mites were extracted from soil samples into 75% ethanol using Berlese’s funnels with electric lamps in the laboratory. Thereafter, the specimens were mounted temporarily in lactic acid in cavity slides for making measurements and illustrations. The identification and illustration of the specimens were performed under a phase-contrast microscope Olympus BX50, equipped with a drawing attachment.

For microscopic imaging, the holotype female was photographed in transmitted light using Canon 5D or Olympus E5 SLR cameras attached to the Olympus BX51 microscope with differential interference contrast (DIC). Obtained frames were stacked and processed using PICOLAY software [2]. For SEM imaging, the specimens were prepared as follows: the mites were air-dried, attached to stubs with double-sided sticky tape, coated in gold and observed in a Zeiss Evo 40 Scanning Electron Microscope.

The morphological terminology of the description follows that of Grandjean (referenced by [3]; overview by [4]). Moreover, some terms and formulas follow [5]. The body measurements are given in micrometers. The prodorsum was measured in lateral view from the tip of the rostrum to the posterior edge; the notogaster was measured as a maximum length in lateral view. The width of the body was expressed as the maximum measurement in the dorsal aspect. The subcapitulum, genitoaggenital and anoadanal plates were measured on the ventral side. Similarly, the lengths of the body setae were measured in the lateral view.

The following abbreviations are used in the description: *ro*, *le*, *in*, *ex*—rostral, lamellar, interlamellar and exobothridial setae; *tr*—trichobothrium; *c*, *d*, *f*, *h*, *ps*—notogastral setae; *ia*, *im*—notogastral lyrifissures; *h*—subcapitular seta; *g*, *ag*, *an*, *ad*—genital, aggenital, anal and adanal setae; *iad*—adanal lyrifissure; and *d*, *l*, *v*—leg setae.

The holotype and paratype were deposited in the Department of Animal Taxonomy and Ecology, the Adam Mickiewicz University in Poznan, Poland.

## 3. Results

**Systematics**

**Figure 1:**
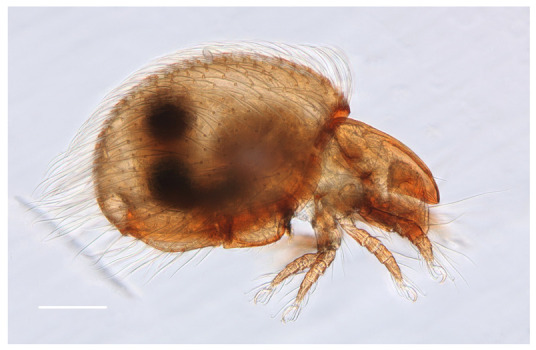
*Protophthiracarus afthonos* Niedbała sp. nov. holotype (DIC), habitus—side view. Scale bar 200 µm.

**Figure 2:**
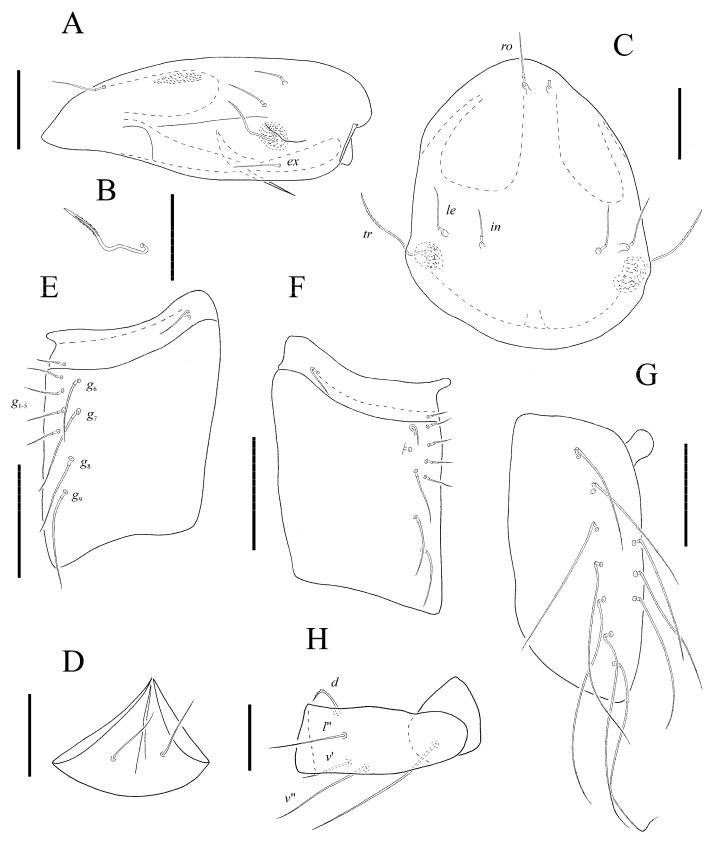
*Protophthiracarus afthonos* Niedbała sp. nov. holotype, (**A**) prodorsum, lateral view; (**B**) trichobothrium, lateral view; (**C**) prodorsum, dorsal view; (**D**) mentum of subcapitulum; (**E**) left genitoaggenital plate; (**F**) right genitoaggenital plate; (**G**) right anoadanal plate; (**H**) trochanter and femur of leg I. Scale bars 100 µm (**A**,**C**,**E**–**G**), 25 µm (**B**,**D**,**H**).

**Figure 3:**
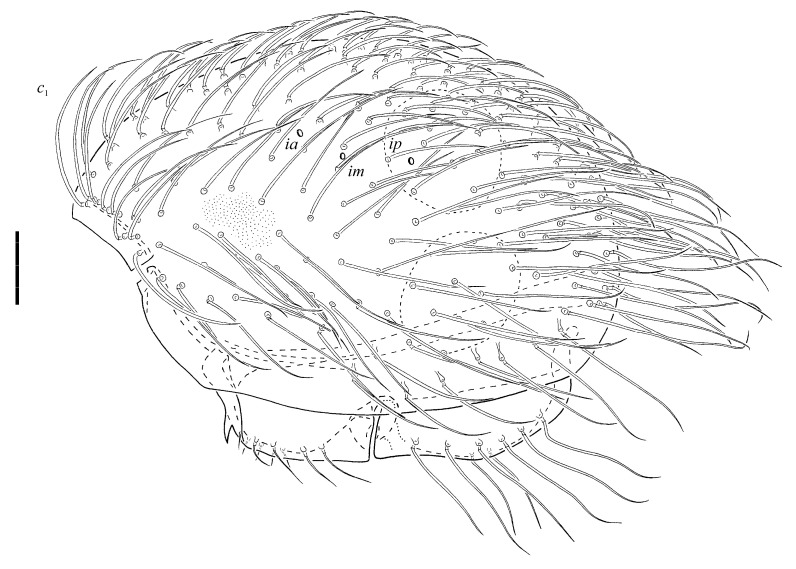
*Protophthiracarus afthonos* Niedbała sp. nov. holotype, opisthosoma, lateral view. Scale bar 100 µm.

**Figure 4:**
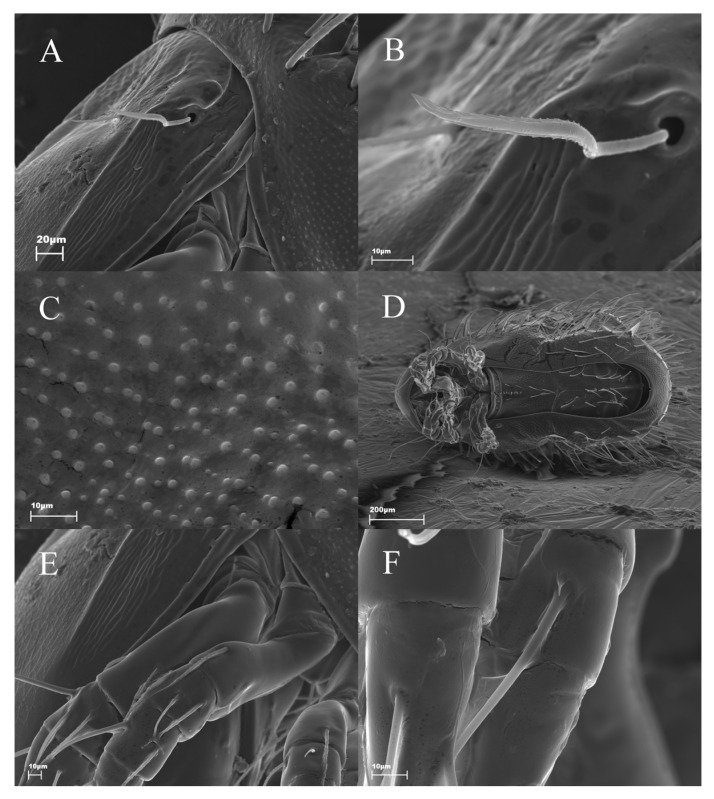
*Protophthiracarus afthonos* Niedbała sp. nov. paratype (SEM); (**A**) part of prodorsum and anterior part of notogaster, lateral view; (**B**) trichobothrium, lateral view; (**C**) notogaster surface texture, lateral view; (**D**) habitus, ventral side; (**E**) parts of prodorsum and legs I and II, lateral view; (**F**) tibia of leg IV, dorsal view.

**Figure 5:**
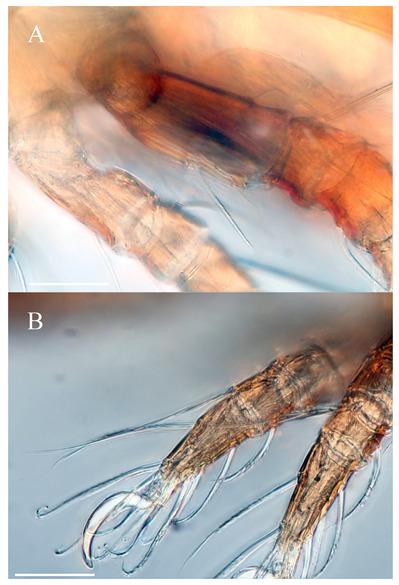
*Protophthiracarus afthonos* Niedbała sp. nov. holotype (DIC); (**A**) parts of legs I and II lateral view; (**B**) parts of leg III and IV. Scale bars 100 µm.

*Protophthiracarus afthonos* Niedbała sp. nov. (Figure 1, Figure 2, Figure 3, Figure 4 and Figure 5)

Description. Measurements of the holotype: prodorsum: length 414, width 293, height 151; setae: *tr* 88, *in* 38, *le* 51, *ro* 63, *ex* 56; notogaster: length 808, width 586, height 545, seta *c*_1_ 268, left genitoaggenital plate 186 × 136, right genitoaggenital plate 202 × 141, anoadanal plate 303 × 146; length of plates measured from the side: genitoaggenital 182, anoadanal 308. Paratype: prodorsum: length 429, height 162; notogaster: length 909, height 616.

Rather large species with strong neotrichy of notogaster (Figure 1), weaker adanal setae. Color: light brown. The surface of the body is punctated (spaced regular mounds visible under high magnification—Figure 4C). Setae finely serrate.

Prodorsum (Figure 2A,C and Figure 4A) with weak lateral carinae. Sigillar fields narrow and well-marked. Posterior furrows absent. Trichobothria (Figure 2B and Figure 4B) long, filiform but rigid, with a clear inner core, distinctly serrate and pointed distally. Interlamellar, lamellar and rostral setae short, spiniform rough: lamellar and interlamellar procumbent; rostral semierect; *tr* > *ro* > *ex* > *le* > *in*.

Notogaster (Figure 3) with ca., 166 pairs of very long (*c*_1_ > *c*_1_–*d*_1_) serrated setae, generally rigid but more flexible towards tips. Due to the huge number of setae, it is extremely difficult to distinguish the setae of appropriate rows. Vestigial setae invisible. Three pairs of lyrifissures (Figure 3), *ia*, *im* and *ip* present.

Venter (Figure 4D). Setae *h* of subcapitulum considerably longer than the distance between them (Figure 2D). Genital setae formula of right genitoaggenital plate (Figure 2F): 5 + 4:1; formula of left plate (Figure 2E): 4 + 4:1; setae *g*_1–5_ shorter than setae *g*_6–9_. In paratype, the formula of both genitoaggenital plates: 4 + 4:1. Anoadanal plates (Figure 2G) with three pairs of setae at their paraxial border (in a position of anal setae) and seven pairs of setae remote from the border (in a position of adanal setae). Setae long, filiform, weakly serrate, slightly more than notogastral setae.

Legs (Figure 2H, Figure 4E,F and Figure 5). Chaetome complete. Setae *d*, *l*″ and *v*′ slightly remote from the anterior end of its segment and situated almost at the same level; seta *v*″ situated posteriorly.

Type material. Holotype and paratype: South America, Peru, Central Peru, Andes, 09°42′58″ S 75°05′33″ W, Huánuco Department, Huánuco Province, Chinchao District, NW Tunel de Carpish, 2770 m a.s.l.; upper soil and leaf litter in primary mountain forest, Winkler extraction, 14 April 2016; leg. S. Friedrich, F. Wachtel and D. Hauth.

Etymology. The specific epithet is derived from Greek *afthonos* meaning “abundant” and refers to the polytrichy of the notogastral setae.

Comparison and diagnosis. This species is distinguishable by the huge neotrichy of the notogastral setae. Multiplication of a number of setae applies to all rows on notogaster, namely: *c*, *d*, *e*, *h* and *ps*. In other species revealing notogastral neotrichy, the enlarged number (multiplication) of setae applies typically to the setae of rear notogastral rows, usually *h* and *ps*.

**A systematic list of ptyctimous mite species (Oribatida) of Peru**

Synonymes are only supplied for species included in the literature cited in this paper; for a complete list of the synonymies of species recorded in Peru, see Niedbała 1992 and 2004. Species names are supplied with a biogeographic element epithet. Species described from Peru as new are marked with asterisk (*). Spellings and collocations of taxonomic names follow the recent catalogue by Niedbała and Liu [6].

**Enarthronota Grandjean, 1947**

**Hypochthonioidea Berlese, 1910**

**Mesoplophoridae Ewing, 1917**

*Mesoplophora* Berlese, 1904

   *Mesoplophora* subgen. nom.

*- bacilla* Niedbała, 2004; neotropical

*- hauseri* Mahunka, 1982; neotropical

-* quasigaveae Niedbała, 2016; endemic

-* sparsa Niedbała, 2004; endemic

   *Parplophora* Niedbała, 1985

-* *subtilis* Niedbała, 1981; pantropical

**Mixonomata Grandjean, 1969**

**Euphthiracaroidea Jacot, 1930**

**Oribotritiidae Balogh, 1943**

*Oribotritia* Jacot, 1924

   *Oribotritia* subgen. nom.

- *didyma* Niedbała et Schatz, 1996; neotropical

*Mesotritia* Forsslund, 1963 (=*Perutritia* Märkel, 1964)

-* *amazonensis* (Märkel, 1964); endemic

-* *curviseta* (Hammer, 1961); neotropical

*Indotritia* Jacot, 1929

   *Indotritia* subgen. nom.

- *bellingeri* Niedbała et Schatz, 1996; pantropical

- *krakatauensis* (Sellnick, 1923) (=*Indotritia acanthophora* Märkel, 1964); pantropical

**Euphthiracaridae Jacot, 1930**

*Acrotritia* Jacot, 1923

-* *clavata* (Märkel, 1964); nearctic and neotropical

- *dikra* (Niedbała et Schatz, 1996); nearctic and neotropical

-* *peruensis* (Hammer, 1961); neotropical

- *refracta* (Niedbała, 1998); pantropical

- *vestita* (Berlese, 1913) (=*Rhysotritia comteae* Mahunka, 1983); pantropical

*Microtritia* Märkel, 1964

-* *tropica* Märkel, 1964; pantropical

**Phthiracaroidea Perty, 1841**

**Phthiracaridae Perty, 1841**

*Phthiracarus* Berlese, 1920

- *anonymus* Grandjean, 1933; semicosmopolitan

- *boresetosus* Jacot, 1930; semicosmopolitan 

-* *helluonis* (Niedbała, 1982); endemic

- *nitens* (Nicolet, 1855); palaearctic (likely introduced)

-* *octosetosus* Niedbała, 2004; endemic

**Steganacaridae** Niedbała, 1986

*Hoplophthiracarus* Jacot, 1933

-* *incredibilis* (Niedbała, 1982); endemic

*Steganacarus* Ewing, 1917

   *Rhacaplacarus* Niedbała, 1986

-* *stenodes* Niedbała, 2004; neotropical

*Protophthiracarus* Balogh, 1972

-* *ventosus* (Hammer, 1961); endemic

-* *afthonos* sp. nov.; endemic

*Notophthiracarus* Ramsay, 1966

-* *fornicarius* (Niedbała, 1982); neotropical

-* *improvisus* (Niedbała, 1982); endemic

-* *inauditus* (Niedbała, 1982); neotropical

*Austrophthiracarus* Balogh et Mahunka, 1978

-* *excellens* (Niedbała, 1982); endemic

*Arphthicarus* Niedbała, 1994

- *inelegans* (Niedbała, 1986); pantropical

-* *simplex* Niedbała, 2017; endemic

*Atropacarus* Ewing, 1917

   *Hoplophorella Berlese, 1923*

- *andrei* (Balogh, 1958); pantropical

- *hamatus* (Ewing, 1909); semicosmopolitan

- *lanceosetus* (Balogh et Mahunka, 1981) (=*Hoplophorella neglecta* Niedbała, 1984, *H. neglectus* Niedbała, 1992); neotropical

-* *stilifer* (Hammer, 1961); pantropical

- *vitrinus* (Berlese, 1913) (=*Hoplophorella scapellata* Aoki, 1965, *H. africana* Wallwork, 1967); semicosmopolitan.

**List of localities and species of ptyctimous Oribatida found in Peru.**

**Figure 6:**
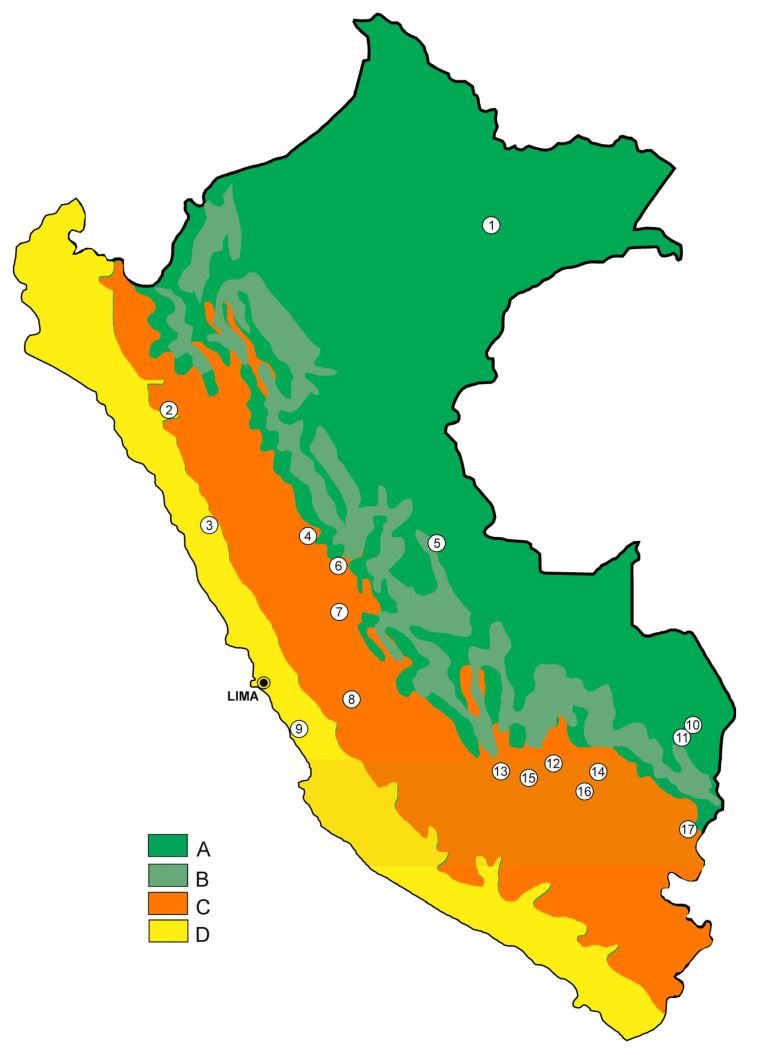
Map of distribution of Peruvian species of ptyctimous oribatid mites plotted against main ecoregions: A—tropical rain forest, B—mountain rain forest, C—mountain grass, scrub and alpine wastes, D—deserts (after [1]; changed). Numbers in circles represent major localities corresponding to those in braces in the “List of localities and species of ptyctimous Oribatida found in Peru”.

The list contains standardized data, with corrections of apparent mistakes in primary sources, modern transliterations of geographic names and the numbers of collected individuals of each species recorded. In addition, it follows the original format as much as possible. Biogeographic distribution is depicted in Figure 6, with locations marked by numbers in braces below.

**Loreto Region**

{1} 

Amazon Basin, Muyuy Island near Iquitos, 105 m a.s.l., lowland rainforest area, jungle, litter, 1956/57, leg. F. Schaller. *I. (I.) krakatauensis—*1 [7].

**Cajamarca Region**

{2} 

At Cajamarca, ca. 7° S, 3000 m a.s.l., in low cushion with stiff leaves on dry moldering soil, below an agave hedge, 5–6 October 1957, leg. M. Hammer. *A. (H.) stilifer*—2 [8].

At Cajamarca, ca. 7° S, 3000 m a.s.l., in almost dry moldering soil with grass, white clover and *Equisetum* below agave and bramble, near the river, 5–6 October 1957, leg. M. Hammer. *P. ventosus*—1 [8].

At Cajamarca, ca. 7° S, 3000 m a.s.l., in dry moss on sun-dried moldering soil below a hedge of agave, 5–6 October 1957, leg. M. Hammer. *A. peruensis*—1 [8].

**Ancash Region**

{3}

Cordillera Blanca, Llanganuco valley, Huascaran National Park, 4000 m a.s.l., litter from bushes and tree *Schinus* sp., 24 August 1976, leg. J. Michejda. *N. fornicarius*—18 [9].

Cordillera Blanca, Llanganuco valley, Huascaran National Park, 3000 m a.s.l., *Schinus* sp., exp. S, 24 August 1976, leg. J. Michejda. *M. curviseta*—2; *A. vestita*—1 [10].

Cordillera Blanca, Llanganuco valley, Huascaran National Park, near Huapi, *Eucalyptus* forest, very dry, 26 August 1976, leg. J. Michejda. *A. vestita*—6 [10].

Cordillera Blanca, Llanganuco valley, Huascaran National Park, near Huapi, *Eucalyptus* forest, wet sample, 26 August 1976, leg. J. Michejda. *A. vestita*—1 [10].

**Huanuco Region**

{4}

Mountain forest area near Tingo Maria, 780 m a.s.l., leaf litter from rubber tree plantation; 1956/57, leg. F. Schaller. *A. clavata*—No. of specimens not given [7].

Mountain forest area near Tingo Maria, 780 m a.s.l., jungle litter; 1956/57, leg. F. Schaller. *M. tropica*—1 [7].

Environments of Tingo Maria, humus on the rocks, decayed wood, and soil (originally three separate samples); 1956/57, leg. F. Schaller. *M. tropica*—No. of specimens not given [7].

{5}

Puerto Inca Province, Yuyapichis District, Área de Conservación Privada Panguana (biological field station), near Rio Yuyapichis (river), 230 m. a.s.l., primary evergreen lowland rainforest, upper soil and leaf litter, Winkler extraction, 09°37′ S, 74°56′ W, 1–21 May 2015, leg. S. Friedrich and F. Wachtel. *A. simplex*—2; *M. (M.) quasigaveae*—86; *M. curviseta*—5; *I. (I.) bellingeri*—2; *A. vestita*—1; *A. (H.) andrei*—2 [11].

Puerto Inca Province, Yuyapichis District, Área de Conservatión Privada, Panguana (biological field station), near Rio Yuyapichis (river), 230–260 m a.s.l., primary evergreen lowland rainforest, upper soil and leaf litter, Winkler extraction, 09°37′ S, 74°56′ W, 20 September–7 October 2013, leg. S. Friedrich and F. Wachtel. *M. (M.) quasigaveae*—41; *M. curviseta*—8; *O. (O.) didyma*—5; *A. clavata—*1; *A. dikra*—19; *A. refracta*—4; *M. tropica*—1; *A. inelegans*—49; *A. (H.) andrei*—7; *A. (H.) hamatus*—7 [12]. 

Puerto Inca Province, Yuyapichis District, Área de Conservación Privada, Panguana (biological field station), near Rio Yuyapichis (river), 230–260 m a.s.l., primary evergreen lowland rainforest, upper soil and leaf litter, 09°37′ S, 74°56′ W, 23 April—9 May 2016, leg. S. Friedrich, F. Wachtel and D. Hauth. *M. (M.) quasigaveae*—51; *A. clavata*—2; *A. refracta*—5; *A. inelegans*—5; *A. (H.) andrei*—9 [13].

{6}

Huánuco Province, Chinchao District, NW Tunel de Carpish, 2770 m a.s.l., 09°42′58″ S 75°05′33″ W, upper soil and leaf litter in primary mountain forest, Winkler extraction, 14 April 2016; leg. S. Friedrich, F. Wachtel and D. Hauth. *P. afthonos* n. sp.—2. 

**Oxapampa Region**

{7}

Near Oxapampa, Rio Esperanza, 2000 m a.s.l., foggy forest area, leaf litter (two separate samples are listed with same collection data in original text); 1956/57; leg. F. Schaller. *M. tropica*—No. of specimens not given; *A. clavata*—8 [7].

Rio Esperanza, 2150 m a.s.l., moss from tree plantation at height of 2 m; 1956/57; leg. F. Schaller. *A. clavata*—No. of specimens not given [7].

Rio Esperanza, 2150 m a.s.l., tree plantation, leaf litter; 1956/57; leg. F. Schaller. *A. clavata*—No. of specimens not given [7].

Rio Esperanza, 2200 m a.s.l., primary forest on steep slope, rotten wood; 1956/57; leg. F. Schaller. *A. clavata*—No. of specimens not given [7].

**Junin Region**

{8}

At Huancayo, ca. 12° S, 3550 m a.s.l., in moist–wet low moss on a vertical slope, always shaded, 2 November 1957, leg. M. Hammer. *A. peruensis*—1 [8].

At Huancayo, ca. 12° S, 3550 m a.s.l., in wet liverworts and moss, shaded, 2 November 1957, leg. M. Hammer. *A. peruensis*—2 [8].

{9}

La Huerta (habitat and substrate unknown), 24–28 November 1955, leg. L. Peña. *A. peruensis*—3; *N. fornicarius*—3 [10].

**Madre de Dios Region**

{10} 

Tambopata National Reserve, ex litter along river, 25 October 1982, L. E. Watrous and G. Mazurek. *A. peruensis*—11; *M. tropica—*10; *A. (H.) vitrinus—*1 [10].

Tambopata National Reserve, ex rotten palm flowers, 28 October 1982, leg. L. E. Watrous and G. Mazurek. *A. (H.) vitrinus—*2 [10].

Tambopata National Reserve, ex bamboo litter, 28 October 1982, leg. L. E. Watrous and G. Mazurek. *M. (M.) sparsa—*1; *M. curviseta—*3; *I. (I.) bellingeri*—3 [10].

{11}

Rio Madre de Dios basin, Puerto Maldonado, 220 m a.s.l., rainforest, 1956/57, leg. F. Schaller. *M. amazonensis*—2 [7].

Rio Madre de Dios, near Puerto Maldonado (“Maldano” in original text, very likely typo), 250 m a.s.l., rain forest (two separate samples but same data); 1956/57, leg. F. Schaller. *M. tropica*—No. of specimens not given [7].

Rio Madre de Dios, Puerto Maldonado, litter under logs at farm pen, 5 September 1976, leg. J. Michejda. *A. (H.) vitrinus*—1 [5].

Rio Madre de Dios, Puerto Maldonado, forest near airport, 5 September 1976, leg. J. Michejda. *A. (H.) lanceosetus*—1 [5].

Rio Madre de Dios, Puerto Maldonado, 500 m a.s.l., wood dust from log laying at a farm pen, 3 September 1976, leg. J. Michejda. *M. subtilis—*6 [14],—8 [10]; *A. (H.) lanceosetus—*3 [15]; *A. (H.) vitrinus*—6 [5].

**Cusco Region**

{12}

Pillahuata, Manu road, 128 km, ex litter in dry streambed, 18 September 1982, leg. L. E. Watrous and G. Mazurek. *M. (M.) bacilla*—1; *M. curviseta*—4; *A. vestita*—11 [10].

Pillahuata, Manu road, 128 km, ex leaf litter, 27 September 1982, leg. L. E. Watrous and G. Mazurek. *M. (M.) bacilla*—3; *M. curviseta*—8; *A. vestita*—6 [10].

Pillahuata, Manu road, 128 km, ex damp leaf litter; 26 September 1982, leg. L. E. Watrous and G. Mazurek. *M. (M.) bacilla*—2; *M. curviseta*—12; *A. vestita*—7; *P. helluonis*—1; *P. octosetosus*—1 [10].

Pillahuata, Manu road, 128 km, ex litter under ferns, 16 September 1982, leg. L. E. Watrous and G. Mazurek. *M. curviseta*—3; *A. vestita*—11 [10].

Pillahuata, Manu road, 128 km, ex litter at seepage area 17 September 1982, leg. L. E. Watrous and G. Mazurek. *M. curviseta*—7; *A. vestita—*1 [10].

Pillahuata, Manu road, 128 km, ex moss and litter on xeric slope, 26 September 1982, leg. L. E. Watrous and G. Mazurek. *M. (M.) bacilla*—1; *A. vestita*—2 [10].

Pillahuata, Manu road, 128 km, ex litter under grass clumps, 16 September 1982, leg. L. E. Watrous and G. Mazurek. *M. curviseta*—1; *A. peruensis*—2 [10].

Pillahuata, Manu road, 128 km, ex rotten logs, 26 September 1982, leg. L. E. Watrous and G. Mazurek. *M. curviseta*—7; *A. peruensis*—3; *S. (R.) stenodes—*1 [10].

Pillahuata, Manu road, 128 km, ex leaf litter after rain, 17 September 1982, leg. L. E. Watrous and G. Mazurek. *M. curviseta*—19; *A. vestita*—4; *P. helluonis*—1 [10].

Pillahuata, Manu road, 128 km, ex litter along stream, 26 September 1982, leg. L. E. Watrous and G. Mazurek. *M. (M.) bacilla*—1; *M. curviseta*—6; *A. vestita*—2 [10].

Consuelo, Manu road, 165 km, ex rotten palm, 5 October 1982, leg. L. E. Watrous and G. Mazurek. *M. hauseri*—2 [10].

{13}

At foot of Machu Picchu, valley of Urubamba River, 2700 m a.s.l., in forest, 2 September 1976, leg. J. Michejda. *H. incredibilis—*1 [9]; *P. boresetosus—*18; *N. fornicarius—*1; *N. inauditus—*10 [5].

Machu Picchu, valley of Urubamba river, near track, 2700 m a.s.l., forest, 2 September 1976, leg. J. Michejda. *A. vestita*—50; *M. curviseta*—4; *P. anonymus*—4 [10].

Urubamba valley, 2700 m a.s.l., dry litter, 3 September 1976, leg. J. Michejda. *A. vestita*—3 [10].

Urubamba valley, Cuscichaca river, 2485 m a.s.l., cloud forest, decayed stump, 30 September 1982, leg. J. Sale. *A. dikra*—19; *P. boresetosus*—3; *P. nitens*—1 [10].

{14}

Rio Madre de Dios basin, near Quince Mil, 650 m a.s.l., mountain forest area, epiphytes, 1956/57, leg. F. Schaller. *A. clavata*—No. of specimens not given [6].

Rio Madre de Dios, near Quince Mil, 650 m a.s.l., in mountain forest, 1956/57, leg. F. Schaller. *M. tropica*—No. of specimens not given [7].

{15}

At the pass Cusco—Pisac, ca. 13°30′ S, 3750 m a.s.l., in moist 2–3 cm high moss between ankle-deep heathery shrubs, 5 February 1955, leg. M. Hammer. *A. peruensis*—1 [8].

Foot of Machu Picchu, ca. 13° S, 2200 m a.s.l., in wet moss on a vertical cliff wall, 1 February 1955, leg. M. Hammer. *M. curviseta*—2; *A. peruensis*—2 [8].

Foot of Machu Picchu, ca. 13° S, 2200 m a.s.l., in wet *Selaginella* sp. on the ground below meter-high vegetation, 1 February 1955, leg. M. Hammer. *M. curviseta*—1; *A. peruensis*—1 [8].

Machu Picchu, exp. N, 3400 m a.s.l., bamboo forest, wet, 1 September 1976, leg. J. Michejda. *P. boresetosus—*3; *A. excellens*—1; *M. curviseta*—1; *A. vestita*—4 [10].

Machu Picchu, 3400 m a.s.l., litter under dense shrubs among ruins, 1 September 1976, leg. J. Michejda. *A. excellens*—1; *P. boresetosus—*3; *N. inauditus*—7 [5].

Machu Picchu, 3600 m a.s.l., tropical rain forest, 1 September 1976, leg. J. Michejda. *M. curviseta*—3; *A. vestita*—10 [10].

Near tourist trail from Wiñay Wayna towards Machu Picchu, 3600 m a.s.l., litter in tropical forest, 1 September 1976, leg. J. Michejda. *A. excellens*—8; *P. helluonis*—4; *N. improvisus*—1; *N. inauditus*—6 [16].

Near tourist trail from Wiñay Wayna towards Machu Picchu, 3400 m a.s.l., litter in tropical forest, E exposure, 1 September 1976, leg. J. Michejda. *A. excellens*—2; *N*. *inauditus—*3 [5].

Near tourist trail from Wiñay Wayna towards Machu Picchu, 3600 m a.s.l., in forest near railroad, 1 September 1976, leg. J, Michejda. *P. boresetosus*—1; *N. inauditus*—1; *P. anonymus*—1 [5].

Machu Picchu, forest near track, 2 September 1976, leg. J. Michejda. *P. anonymus*—1; *P. boresetosus*—1 [10].

Machu Picchu, rain forest, exp. E, 3400 m a.s.l., 2 September 1976, leg. J. Michejda. *A. vestita*—8 [10].

{16}

Marcapata, road to Puerto Maldonado, km 175, ex leaf litter, 21 October 1982, leg. L. E. Watrous. *M. curviseta*—2; *P. helluonis*—1 [10].

**Puno Region**

{17}

At Sillustani, north of Puno, ca. 15° S, 3900 m a.s.l., almost dry moss on a vertical slope below shrubs, shaded, 16 November 1957, leg. M. Hammer. *M. curviseta*—1 [8].

The mite material was collected from soil (below an agave hedge, dry moldering soil with grass, humus on the rocks), litter (under ferns, at seepage areas, under grass clumps, from bushes and trees, in a bamboo forest, in a tropical rain forest, under trunks, in dry streambed, damp leaf litter, stiff leaves on dry moldering), moss, liverworts and clubmoss (between ankle-deep heathery shrubs, wet and dry moss on a vertical cliff-wall, wet moss on a vertical cliff, wet liverworts and moss, wet *Selaginella* on the ground), decomposing plant matter (rotten palm flowers, decayed wood, wood dust from a fallen tree trunk, rotten logs, rotten palms, decayed stump in a cloud forest) and other living plant matter (white clover and *Equisetum* below agave and bramble, epiphytes from mountain forest).

**Key to ptyctimous Oribatida of Peru**

1. Genital and anal openings well separated ........................... Enarthronota ........................ 2-. Genital and anal openings joined .................................. Mixonomata ................................. 62. Three pairs of anal setae ........................................................................... *Mesoplophora*
*(Parplophora)* ........................................................................ *M. (P.) subtilis* (Figures 1–24 in [14])-. Two pairs of anal setae .............................. *Mesoplophora (Mesoplophora)* ............................. 33. “Notogastral” setae smooth, formula of genital setae 5:2 ...................................................... .................................................................................................. *M. (M.) bacilla* (Figure 3F–I in [10])-. “Notogastral” setae spinose or rough, formula of genital setae 6:1 ................................... 44. Trichobothria with slightly fusiform head covered with nine small setae .......................... .............................................................. *M. (M.) hauseri* (Figures 1–3 in [17]; Figure 4H–K in [10])-. Trichobothria setiform covered with more than ten small setae ........................................ 55. Anal setae *an*_1_ located in posterior half of plates; trichobothria covered with 11–12 pairs of small setae ............................................................. *M. (M.) quasigavae* (Figures 12–22 in [12])-. Anal setae *an*_1_ located in anterior half of plates; trichobothria covered with 15 pairs of small setae ............................................................................ *M. (M.) sparsa* (Figure 6E–G in [10])6. Body considerably compressed laterally, anogenital region narrow, V-shaped ................ .................................................... Euphthiracaroidea ................................................................... 7-. Body less compressed laterally, anogenital region relatively wide, U-shaped ................... .................................................... Phthiracaroidea ...................................................................... 177. Ventral plates not completely fused, at least anal plates separated by suture, longitudinal suture of ano-genital region without interlocking triangle .................................. ............................................... Oribotritiidae ................................................................................. 8-. Ventral plates completely fused, at least one triangle in longitudinal suture of ano-genital region present ....................................... Euphthiracaridae ........................................................ 128. Genitoaggenital suture incomplete, two plates well delineated from each other only posteriorly ................................................. *Indotritia (Indotritia)* ................................................ 9-. Genitoaggenital suture complete .......................................................................................... 109. Interlamellar setae almost as the half of height of prodorsum, not bent distally, exobothridial setae well developed ................................ *I. (I.) bellingeri* (Figures 34–49 in [18])-. Interlamellar setae fine, no longer than one fourth of height of prodorsum, bent distally, exobothridial setae vestigial .................................... *I. (I.) krakatauensis* (Figure 18M–O in [10])10. Bothridial scale situated above bothridium, scisure between genital and anal plates present .................... *Oribotritia (Oribotritia)* .................... *O. (O.) didyma* (Figures 7–14 in [18])-. Bothridial scale situated below bothridium, scisure between genital and anal plates absent ............................................................ *Mesotritia* .......................................................................... 1111. Rostral setae situated distinctly anteriorly of lamellar setae, adanal setae *ad*_1_ and *ad*_2_ similar in length ............................. *M*. *amazonensis* (Figure 6A–G in [7]; Figure 13A–H in [10])-. Rostral setae situated at the level with lamellar setae, adanal setae *ad*_1_ considerably longer than setae *ad*_2_ .................... *M*. *curviseta* (Figures 134–134C in [8]; Figure 15A–I in [10])12. Genitoaggenital plates with 4–6 genital setae .......................................................*Microtritia* ........................................................................... *M*. *tropica* (Figure 11 in [7]; Figure 35E–H in [10])-. Genitoaggenital plates with 7–9 genital setae .......................... *Acrotritia* .......................... 1313. Two pairs of lateral prodorsal carinae present on each side ........................................... 14-. One single pair of lateral prodorsal carinae present on each side ..................................... 1514. Nine pairs of genital setae, one pair in progenital position; tarsi of legs monodactylous ................................................................................................ *A*. *refracta* (Figures 155–159 in [19])-. Nine pairs of genital setae, all in genital position; tarsi I bi-, tarsi II-IV tridactylous ............ ....................................................... *A*. *peruensis* (Figures 133, 133A in [8]; Figure 32M–O in [10])15. Lateral carinae of prodorsum not forked distally ................................................................. .................................................................. *A*. *clavata* (Figures 16A–C in [7]; Figures 29E–I in [10])-. Lateral carinae of prodorsum forked distally ....................................................................... 1616. Trichobothria setiform ..................................................... *A*. *dikra* (Figures 111–117 in [18])-. Trichobothria with distinct fusiform head ............................................................................... ..................................... *A*. *vestita* (= *Rhysotritia comteae*: Figures 4–7 in [20]; Figure 29J–L in [10])17. Setae smooth, fine, attenuate, tapering to distal end ................................. Phthiracaridae ................................................................. *Phthiracarus* ................................................................ 18-. Setae (except exobothridial) rough or covered with small spines of different shapes but not smooth and attenuate ....................................... Steganacaridae ....................................... 2218. Trichobothria long and narrow, their length more than 10 times of width .................. 19-. Trichobothria short and wide, length not more than 10 times of width .......................... 2019. Neotrichy of setae present, notogaster with 16 pairs of setae, anoadanal plates with six pairs of adanal setae ............................................................ *P*. *octosetosus* (Figure 39A–F in [10])-. Neotrichy of setae absent, notogaster with 15 pairs of setae, anoadanal plates with three pairs of adanal setae ......................................................................................................................... .................... *P*. *boresetosus* (Figures 15–17 in [21]; Figures 517–522 in [22]; Figure 36I-O in [10])20. Neotrichy present, 21–28 pairs of notogastral setae, three to four pairs anal and six to nine pairs of adanal setae present ...................................... *P*. *helluonis* (Figures 22–43 in [16])-. Neotrichy absent, always 15 pairs of notogastral setae, two pairs of anal and three pairs of adanal setae present ............................................................................................................... 2121. Four pairs of lyrifissures, *ia*, *im*, *ip*, *ips* present, adanal setae *ad*_1_ and *ad*_2_ vestigial ............ ...................................................................... *P*. *nitens* (Figures 1–6 in [23]; Figures 38M–T in [10])-. Two pairs of lyrifissures, *ia* and *im* present, adanal setae well developed ............................. .................................................. *P*. *anonymus* (Figures 1A,B; 2A–C in [24]; Figure 36E–H in [10])22. Three setae (*ad*_1_, *an*_1,_
*an*_2_) in a row near paraxial margin of anoadanal plate ................. 23-. Two setae (*an*_1_ and *an*_2_) near paraxial margin of anoadanal plate ..................................... 3023. Setae *d* on tibiae IV long, independent of solenidia ...................................................
*Steganacarus* (*Rhacaplacarus*) .............................................. *S*. (*R*.) *stenodes* (Figure 52F–J in [10])-. Setae *d* on tibiae IV short, coupled with solenidia ............................................................... 2424. Genital setae *g*_7-9_ displaced towards paraxial margin of genitoaggenital plates and arranged in a row with setae *g*_1-5_, setae *g*_6_ not displaced ............. *Protophthiracarus* ............ 25-. All genital setae located in a row along paraxial margin ........................................................ ........................ *Atropacarus (Hoplophorella)* ................................................................................ 2625. Notogaster with 17 pairs of short (*c*_1_ < *c*_1_–*d*_1_) setae .................................................................. ........................................................ *P*. *ventosus* (Figures 131, 131A in [8]; Figure 88P–U in [10])-. Notogaster with ca. 166 pairs of long setae; *c*_1_ setae considerably longer than distance *c*_1_- *d*_1_ ........................................................................................................................ *P*. *afthonos* sp. nov.26. Notogastral setae wide, phylliform .................................................................................... 27-. Notogastral setae longer, slightly lanceolate ....................................................................... 2927. Rostral setae directed forwards ................................ *A. (H.) hamatus* (Figure 99D–J in [10])-. Rostral setae directed inwards ............................................................................................... 2828. Notogaster with median band ................................... *A. (H.) andrei* (Figures 11–23 in [25])-. Notogaster without median band ............................. *A. (H.) vitrinus* (Figure 102J–O in [10])29. Notogastral setae longer (*c*_1_ > 1/2*c*_1_–*d*_1_), setae *v’* of femora absent ......................................... ..................................................................................... *A. (H.) lanceosetus* (Figure 100G–T in [10])-. Notogastral setae shorter (*c*_1_ < 1/2*c*_1_–*d*_1_), setae *v’* of femora present ...................................... ................................................ *A. (H.) stilifer* (Figures 132–132C in [8]; Figure 101O–T in [10])30. Setae *d* on tibiae of legs IV long, independent of solenidia .................................................. ........ *Hoplophthiracarus* .......................................................... *H*. *incredibilis* (Figures 1–16 in [9])-. Setae *d* on tibiae IV short, coupled with solenidia ............................................................... 3131. Genital setae arranged in a row near paraxial margin of plates .......................................... ....................................... *Notophthiracarus* .................................................................................. 32-. Genital setae arranged in two rows; setae *g*_6_ and *g*_7_ remote from paraxial margin or only setae *g*_6_ remote from margin ...................................................................................................... 3432. Majority of notogastral setae hooked distally; two pairs of lyrifissures *ia* and *im* present ............................................................................................... *N*. *improvisus* (Figures 44–61 in [16])-. Notogastral setae not hooked distally; three or four pairs of lyrifissures present .......... 3333. Surface of notogaster punctated; three pairs of lyrifissures *ia*, *im*, *ip* present; formula of genital setae 6:3 .................................................................. *N*. *fornicarius* (Figures 17–35 in [16])-. Surface of notogaster covered with distinct concavities; four pairs of lyrifissures *ia*, *im*,
*ip*, *ips* present; formula of genital setae 5:4 ........................ *N*. *inauditus* (Figures 62–79 in [16])34. Genital setae arranged in two rows; setae *g*_6_ and *g*_7_ remote from paraxial margin .......... ............................ *Austrophthiracarus* ...................................... *A*. *excellens* (Figures 1–21 in [16])-. Only setae *g*_6_ remote from margin, other genital setae forming one row near paraxial margin .......................................................... *Arphthicarus* ......................................................... 3535. Prodorsum with long line in extension of sinus; notogaster with setae *c*_3_ the smallest, spiniform, rough, other setae longer, ciliate, obtuse distally; three pairs of lyrifissures *ia*,
*im*, *ip* present ................................................................................ *A*. *simplex* (Figure 2A–I in [18])-. No line in extension of sinus on prodorsum; notogaster with setae *c*_3_ ad *cp* the smallest and different shape than other notogastral setae; four pairs of lyrifissures *ia*, *im*, *ip*, *ips* present ...................................................................................... *A*. *inelegans* (Figures 1–7 in [26]).

## 4. Discussion

The phenomenon of setal multiplication on the particular body area of mites is known under the name neotrichy: “Néotrichie est la terme générale […] est la formation secondaire de poils, dans un territoire, par la multiplication de poils préexistant dans ce territoire” [27,28]. Neotrichy is well known among phthiracaroid mites, where extra setae appear usually on the notogaster and anoadanal plates. It also sometimes results in hyper-trichy, as exemplified by *P. afthonos* n. sp.

We report a new species of the genus *Protophthiracarus* (Phthiracaroidea) from the Peruvian Andes, which has a high, previously unseen number of notogastral setae. To our best knowledge, this is the second case of such rich neotrichy of notogastral setae ever known. Until now, the most hairy ptyctimous mite species known and probably the most neotrichous oribatid mite ever was *Atropacarus* (*A*.) *niedbalai* from New Zealand [29]. This species shows extreme neotrichy on the prodorsum, notogaster, genitoaggenital and anoadanal plates, whereas the neotrichy of the newly described *Protophthiracarus afthonos* is seen on the notogaster and anoadanal plates only. However, the number of setae on the notogaster being ca. 166 pairs is fairly higher than that of the New Zealand species (109–115 pairs). The neotrichy on the notogaster has a form of plethotrichy because numerous setae are displaced unevenly and asymmetrically arranged.

Neotrichy occurs independently in different phylogenetic linages of phthiracaroid mites [5] and, in the case reported herewith, concerns the taxon belonging to the genus *Protophthiracarus*, which is generally poor in species. It is more frequent in species from the Southern Hemisphere, especially Neotropical and Australasian regions [27,28,29,30].

It is also worth mentioning that the neotrichy itself cannot be considered an argument strong enough to create upper-level taxa [5,27,30].

The genus *Protophthiracarus* was proposed by Balogh [31], with a type species *Notophthiracarus chilensis* Balogh et Mahunka, 1967 [32]. It is well represented in the fauna of the southern hemisphere, except the Australasian region. The genus *Protophthiracarus* comprises 47 described species and 2/3 of them originate from the Neotropical region.

Approximately half of the 36 species (19) known in Peru have been described as new for science. They belong to very different genera: Mesoplophoridae: *Mesoplophora*—3 spp.; Euphthiracaroidea: Oribotriidae: *Oribotritia*—1 sp., *Mesotritia*—1 sp., *Acrotritia*—2 spp., *Microtritia*—1 sp., Phthiracaroidea: Phthiracaridae: *Phthiracarus*—2 spp.; Steganacaridae: *Hoplophthiracarus*—1 sp., *Steganacarus*—3 spp., *Austrophthiracarus*—1 sp., *Arphthicarus*—1 sp., *Notophthiracarus*—3 spp. (see Systematic list). Thus, the species described from Peru represent ptyctimous mites of both main orders of Oribatida, namely Enarthronota and Mixonomata. Out of the abovementioned 36 species, 19 have been described by the senior author (including three with H. Schatz [18]).

All species reveal similar proportions in biogeographic distribution, those being endemites (11 species), neotropical (11 species) and widely distributed ones (semicosmopolitan—4 species, and pantropical—9 species), each group sharing ca. 1/3rd of the pool [6]. Endemic species are generally scarce, possibly except for *Mesoplophora (Mesoplophora) quasigaveae*. Two endemic species are numerously represented in few samples, but each from one region only: *A. excellens* at Machu Picchu, and *M. quasigavae* in the Puerto Inca Province.

The majority of more broadly distributed species occur in a larger number of various localities, e.g., pantropical *A. vestita* in 17, neotropical *M. curviseta* in 20 and *A. peruensis* in 10 localities. The number of individuals is not distribution-dependent, and even though the most numerous are neotropical species, pantropical and endemic species are also numerous, and two pantropical and endemic species are richest in numbers.

Three neotropical species, *A. clavata*, *N. fornicarius* and *N. inauditus,* reveal that Guyanan distribution occurs only in the northern part of the Neotropical region (Antilles, Venezuela, Ecuador, Peru, Bolivia). This may prove that quite specific climatic and environmental factors shape the distribution of ptyctimous mites in Peru.

The reported resulting individual counts were collected from a variety of substrates, ranging from soil and litter through to decayed organic plant matter and to lower vascular and epiphytic plants.

The most numerous ptycimous mites (total number of individuals in samples/share in total number of individuals of all spp.) are as follows: *Acrotritia vestita*—pantropical, 138/18.1%; *Mesoplophora (Mesoplophora) quasigaveae*—endemic, 137/18%; *Mesotritia curviseta*—neotropical, 99/13%; *Notophthiracarus inauditus*—neotropical, 54/7.1%; *Arphthicarus inelegans*—pantropical, 49/6.4%; *Notophthiracarus fornicarius*—neotropical, 41/5.4%; *Acrotritia dikra*—nearctic and neotropical, 38/5%; *Phthiracarus boresetosus*—semicosmopolitan, 37/4.9%; *Acrotritia peruensis*—neotropical, 25/3.3%; *Austrophthiracarus excellens*—endemic, 19/2.5%; *Atropacarus (Hoplophorella) vitrinus*—semicosmopolitan, 17/2.2%. The most numerous species belong to various genera of Mesoplophoridae (one species), Euphthiracaroidea (four species) and Phthiracaroidea (six species).

Most of the geographic localities from where the mite material is reported herewith are within the Andes range and its highland vicinities. This apparently reflects more the attitude of the collectors than the true geographic distribution of the species. Thus, our conclusion is that ptyctimous Oribatida needs more extensive sampling from mountain and highland (and to some extend lowland tropical) areas in order to evaluate its real abundance and density.

## Data Availability

Data sharing not applicable.
